# Extracellular Vesicles Isolated from Human Induced Pluripotent Stem Cell-Derived Neurons Contain a Transcriptional Network

**DOI:** 10.1007/s11064-020-03019-w

**Published:** 2020-05-02

**Authors:** David A. Hicks, Alys C. Jones, Nicola J. Corbett, Kate Fisher, Stuart M. Pickering-Brown, Mark P. Ashe, Nigel M. Hooper

**Affiliations:** 1 Division of Neuroscience and Experimental Psychology, School of Biological Sciences, Faculty of Biology, Medicine and Health, Manchester Academic Health Science Centre, University of Manchester, Manchester, M13 9PT UK; 2 Division of Molecular & Cellular Function, School of Biological Sciences, Faculty of Biology, Medicine and Health, Manchester Academic Health Science Centre, University of Manchester, Manchester, M13 9PT UK; 3 Present Address: Manchester University NHS Foundation Trust, Manchester, UK; 4 Present Address: Discovery Research UK, MSD, London, UK

**Keywords:** Extracellular vesicles, Neurons, RNA seq, Proteomics, Cell signalling

## Abstract

Healthy brain function is mediated by several complementary signalling pathways, many of which are driven by extracellular vesicles (EVs). EVs are heterogeneous in both size and cargo and are constitutively released from cells into the extracellular milieu. They are subsequently trafficked to recipient cells, whereupon their entry can modify the cellular phenotype. Here, in order to further analyse the mRNA and protein cargo of neuronal EVs, we isolated EVs by size exclusion chromatography from human induced pluripotent stem cell (iPSC)-derived neurons. Electron microscopy and dynamic light scattering revealed that the isolated EVs had a diameter of 30–100 nm. Transcriptomic and proteomics analyses of the EVs and neurons identified key molecules enriched in the EVs involved in cell surface interaction (integrins and collagens), internalisation pathways (clathrin- and caveolin-dependent), downstream signalling pathways (phospholipases, integrin-linked kinase and MAPKs), and long-term impacts on cellular development and maintenance. Overall, we show that key signalling networks and mechanisms are enriched in EVs isolated from human iPSC-derived neurons.

**Electronic supplementary material:**

The online version of this article (10.1007/s11064-020-03019-w) contains supplementary material, which is available to authorized users.

## Introduction

Extracellular vesicles (EVs) represent a major contact-independent mechanism for intercellular communication in the brain [1]. EVs comprise a wide range of sizes, cargoes and subcellular origins [2, 3]. Small EVs, defined as having a diameter of approximately 50–150 nm, include exosomes which are of endosomal origin [4, 5]. EVs are released from the cell and contain a heterogeneous population of macromolecules, including lipids, proteins and various classes of RNA [2]. The protein cargo can be transferred to recipient cells, for example being endocytosed by dendritic cells and the cargo presented by MHC class I molecules to CD4^+^ T cells [6]. The mRNA cargo can be translationally competent as demonstrated by microarray analysis in recipient cells [7] and translation of Arc protein in recipient cells [8], which may modulate synaptic function. In addition, EVs have been implicated as a conduit for the cell to cell transfer of several misfolded proteins in neurodegenerative diseases, which may be a mechanism for the spread of pathogenic protein conformers in neurodegeneration [9]. A range of cellular stresses, including hypoxia and hypoglycaemia have been shown to modify EV cargo, including mRNA [10–15].

The biogenesis of small EVs (sEVs) is a complex process, involving a multiplicity of accessory proteins and has been comprehensively reviewed [2, 3]. In brief, as endosomes mature, they fill with intraluminal vesicles to form a multi-vesicular body (MVB) [16]. The biogenesis can be ESCRT-dependent, or independent, driven by tetraspanins and syntenin [2]. Hence ESCRT components and accessories, such as Tsg101 or Alix and tetraspanins (usually CD9, CD63 and CD81) all represent common sEV markers [17], although cell surface tetraspanin localisation has been reported [18]. The contents of the MVB can be degraded by the lysosome or released into the extracellular milieu [16]. The mechanisms driving sEV secretion are not fully understood, but certain Rab proteins (e.g. Rab27), lipids (e.g. ceramide and sphingomyelin) and SNAREs have all been shown to have important roles in the process [19–22].

Despite the potential involvement of EVs in neuronal intercellular communication [23], there is limited information on the cargo within EVs secreted from neurons. In this study we have isolated EVs from human neurons derived from induced pluripotent stem cells (iPSCs). Transcriptomic, proteomics and bioinformatics analyses were then performed on the EVs to identify the molecules and pathways contained within the neuronal sEVs. We show that sEV-enriched mRNAs and proteins are significantly linked to processes involved in the development and maintenance of the nervous system, forming defined signalling networks.

## Material and Methods

### Materials

All chemicals were purchased from Fisher Scientific (Loughborough, Leicestershire, UK) unless otherwise stated.

### Methods

#### Cell Culture

The iPSC line, OX1-19 (obtained from S. Cowley, University of Oxford) [24–26] was maintained on Matrigel (BD Biosciences, Wokingham, Berkshire, UK) in mTeSR1 medium (StemCell Technologies, Cambridge, UK) containing 50 U/ml penicillin and 50 µg/ml streptomycin (Sigma-Aldrich, Gillingham, Dorset, UK) in a humidified incubator at 37 °C in a 5% CO_2_, 95% air atmosphere. Pluripotency and successful cortical neuron differentiation were confirmed using immunofluorescence microscopy with appropriate markers (pluripotency: Sox2, SSEA4, Oct4 and Nanog; mature neuron: MAP2, βIII tubulin, Tbr1 and Sat2b). The iPSCs were differentiated to cortical neurons as described previously [27], using dual-SMAD inhibition by 1 µM dorsomorphin and 10 µM SB431452 (Tocris, Bio-techne). Following successful differentiation, neural progenitor cells were re-plated on day 35 post-induction at 300,000 cells/ well onto poly-ornithine and laminin-coated (Sigma-Aldrich) 6-well polystyrene tissue culture plates (Greiner Bio One, Stonehouse, Glos, UK) and neurons cultured until day 75 + post-induction with media changes every 2–3 days. Post-induction culture medium was 1:1 DMEM F12: neurobasal medium containing B27 and N2 supplements, 2 mM L-glutamine, 100 µM 2-mercaptoethanol, 25 µM insulin, 100 U/mL penicillin, 100 µg/mL streptomycin (all Life Technologies) and 0.5% non-essential amino acids (Sigma-Aldrich) at 200 µl.cm^−2^ culture area. Minimal cell death was observed microscopically during the culture period. Separate inductions from iPSCs to neurons were regarded as biological replicates and n = 3, unless otherwise stated.

#### Immunocytochemistry

iPSCs or iPSC-derived neurons were cultured as described above, then washed three times in PBS before fixation in 4% paraformaldehyde for 10 min. Cells were washed in PBS, then permeabilised in 0.2% Triton X-100 in PBS for 5 min. After further washing, cells were incubated in blocking buffer (10% donkey serum in PBS) in PBS for 3 h at room temperature followed by incubation with primary antibody (overnight, 4 °C). Cells were then washed, incubated with secondary antibody (conjugated to Alexa Fluor 488 (RRID: AB_2556542) or Alexa Fluor 568 (AB_25340); Life Technologies, Paisley, UK), followed by washing in PBS and mounting on slides using DAPI-containing mounting medium (Southern Biotech, Birmingham, AL, USA). Slides were visualised using the Evos FL (Thermo Fisher Scientific, Loughborough, UK). Primary antibodies used were for: Sox2 (AB_2341193), SSEA4 (AB_778073), Oct4 (AB_445175), Nanog (AB_446437), MAP2 (AB_297885), βIII tubulin (AB_444319), Tbr1 (AB_2200219) and Sat2b (AB_882455) (all Abcam, Cambridge, UK).

#### EV Isolation

Conditioned medium from 5 × 10^6^ cells was harvested by pipetting from neuronal cultures (approximately 50 ml total) and centrifuged in polypropylene tubes (300×*g* for 10 min, then 2000×*g* for 20 min at 4 °C with maximum brake). Medium was then filtered through a 0.22 µm filter into a Vivaspin 20 (100 kDa MWCO) centrifugal concentrator, followed by centrifugation at 3900×*g* (4 °C, maximum brake) to reduce the volume to 0.5 ml. The concentrated medium was then added to a qEV original column (Izon Sciences, Oxford, UK) and separated by size exclusion chromatography (SEC). The first six fractions represented the void volume, with vesicles eluted in filtered phosphate-buffered saline (PBS) in subsequent fractions of 0.5 ml. After EV isolation, RNA/ protein cargo was isolated immediately. SEC is considered an intermediate recovery, intermediate specificity technique. Separate inductions from iPSCs to neurons were regarded as biological replicates and n = 3, unless otherwise stated.

#### Electron Microscopy

The vesicular fractions (fractions 7–9) were pooled and centrifuged at 100,000×*g* to pellet the EVss. The samples were fixed with 4% formaldehyde + 2.5% glutaraldehyde in 0.1 M HEPES buffer (pH 7.2). Samples were post-fixed with 1% osmium tetroxide + 1.5% potassium ferrocyanide in 0.1 M cacodylate buffer (pH 7.2) for 1 h, then in 1% uranyl acetate in water overnight. The samples were dehydrated in ethanol infiltrated with Low Viscosity resin (TAAB Laboratory and Microscopy, Aldermaston, Berks, UK) and polymerized for 24 h at 60 °C. Sections were cut with a Reichert Ultracut ultramicrotome and observed with FEI Tecnai 12 Biotwin microscope at 100 kV accelerating voltage. Images were taken with Gatan Orius SC1000 CCD camera. EV diameter was calculated using ImageJ (NIH, USA).

#### Dynamic Light Scattering

Unconcentrated fractions eluted from the qEV column were analysed for particle diameter using the Zetasizer Nano (Malvern Panalytical, Malvern, Worcestershire, UK). Three analyses were performed per sample.

#### Cell Lysis

Cells were washed twice in ice-cold PBS and harvested in PBS. Cells were pelleted at 3000×*g* for 5 min (4 °C) and re-suspended in 6 × volume of lysis buffer (RIPA buffer: 50 mM Tris–HCl (pH 8.0), 150 mM sodium chloride, 1% Igepal CA-630 (Sigma-Aldrich), 0.5% sodium deoxycholate, 0.1% SDS, 1 mM sodium fluoride, 1 mM sodium orthovanadate, and Complete Protease Inhibitor cocktail (Roche Diagnostics, Burgess Hill, West Sussex, UK)). Lysis was performed for 30 min on ice, followed by centrifugation at 3000×*gs* for 30 min (4 °C) to yield the RIPA-soluble fraction as the supernatant, which was used for immunoblotting.

#### SDS-PAGE and Immunoblotting

SEV fractions eluted from the qEV column were concentrated ten-fold with an Amicon 10 centrifugal concentrator and then boiled for 5 min in 5 × SDS-PAGE sample buffer containing DTT (Jena Biosciences, Jena, Germany). Samples were separated by electrophoresis 120 V for 90 min on a polyacrylamide gel containing 10% acrylamide. After SDS-PAGE, proteins were transferred to polyvinylidene fluoride (PVDF) membranes for 75 min at 125 V (Bio-Rad). The PVDF membranes were incubated for 2 h in blocking solution (5% (w/v) milk power, 2% (w/v) BSA in TBS + 1% (v/v) Tween-20 (TBST)) and then incubated overnight in primary antibody (5% (w/v) milk powder in TBS). The PVDF membranes were washed 4 × 10 min with TBST before the addition of secondary antibody (HRP-conjugated anti-IgG; 5% (w/v) milk powder in TBST, 1:5000 (Thermo Fisher Scientific)) for 1 h, followed by 4 × 10 min washes with TBST. Protein bands were visualized and, where appropriate, quantified, by chemiluminescence (Clarity Western ECL Blotting Substrate, Bio-Rad) using a G:BOX and GeneTools software (Syngene, Cambridge, UK). Alternatively, polyacrylamide gels were stained with Coomassie Blue (R-250 Brilliant Blue in 45% methanol, 45% H_2_O, 10% glacial acetic acid) for 30 min and destained for 3 h with 45% methanol, 45% H_2_O, 10% glacial acetic acid. Primary antibodies used were for Tsg101 (1:500, Abcam, Cambridge, UK; RRID: AB_1271357), CD9 (1:100, BioLegend, London, UK; AB_314907), mitofilin (1:500) and Grp78 (1:500, Proteintech, Manchester, UK; AB_2119855), TDP-43 (1:500, Proteintech, Manchester, UK; AB_615042), Src (1:200, Cell Signaling Technology, Leiden, The Netherlands, AB_2106059).

#### RNA-Seq

Vesicular samples (fractions 7–9) were pooled and incubated with 0.4 µg/µl RNase A (Sigma-Aldrich) for 10 min followed by extraction of RNA with the Arcturus PicoPure RNA Isolation Kit (Thermo Fisher) and oligo (dT) primed cDNA synthesis using the SMART-Seq v4 Ultra Low Input RNA Kit for Sequencing (Takara Bio, Saint-Germain-en-Laye, France). Adapter indices were used to multiplex libraries, which were pooled prior to cluster generation using a cBot instrument. The loaded flow-cell was then paired-end sequenced (76 + 76 cycles, plus indices) on an Illumina HiSeq4000 instrument. Finally, the output data was demultiplexed (allowing one mismatch) and BCL-to-Fastq conversion performed using Illumina’s bcl2fastq software, version 2.17.1.14.

#### Bioinformatics

The paired-end RNA-seq reads were quality assessed using FastQC (v 0.11.3), FastQ Screen (v 0.9.2). Mean Phred scores across the short reads were greater than 38. Reads were processed with Trimmomatic (v 0.36) (to remove technical sequences and poor quality bases) In neurons, 13% of bases were removed and in EVs this was 28%. The RNA-seq reads were mapped against the reference human genome (hg38) using STAR (version 2.5.3a). Counts per gene were calculated with STAR using annotation from GENCODE (v27). Normalisation and differential gene expression analysis was performed using DESeq2 (using Benjamini–Hochberg correction for false discovery). The mean library size for neurons was 5 × 10^7^ with a mapping rate of 1.5 × 10^8^ reads per hour. The corresponding figures for EV libraries are 2.5 × 10^7^ and 5.2 × 10^7^. As the reads are different between cells and EVs (i.e. greater in cells, the data were sub-sampled to equalise the number of reads. This did not lead to a significant relative diminution of gene abundance in the cells as determined by linear regression analysis on the original and sub-sampled datasets nor substantial changes between EV and cell samples as determined by principal component analysis. RNA-seq data have been deposited in the ArrayExpress database at EMBL-EBI (www.ebi.ac.uk/arrayexpress) under accession number E-MTAB-8254.

Ingenuity Pathways Analysis (IPA, Qiagen, Hilden, Germany) was used to explore gene networks for hypothesis generation. For mRNA, analysis thresholds were set at log_2_ fold change > 1 and q ≤ 0.05. Core analysis was based on log_2_ fold changes and the IPA knowledge database filtered to only include experimental findings in mammalian systems, limited to brain, primary neurons or neuroblastoma cells. Networks were limited to 70 focus molecules.

Genes were ordered by descending p value and the top 150 queried against UTRdb [28] to establish 5′ and 3′ UTR length. GC content was established using GCevobase [29] (querying 18,104 genes), predicted G quadruplexes using the EuQuad module of Quadbase 2 (target G_x_L_1-y_G_x_L_1-y_G_x_L_1-y_G_x_ where x = 3, y = 7 and L is any base; querying top 1530 genes as organised by descending p value) [30] and poly (A) tail length (querying 3863 genes available from TAIL-seq) from TAIL-seq analysis [31]. Data were analysed by D’Agostino-Pearson test for Gaussian distribution and Spearman’s rank correlation.

#### Quantitative PCR (qPCR)

qPCR reactions were prepared as follows (total 20 µl): 1 µl cDNA, 500 nM each of forward and reverse primers with iQ Supermix (Bio-Rad). Thermal cycler (QuantStudio 3, Applied Biosystems, Thermo Fisher Scientific) parameters were set as follows: 3 min @ 95 °C and forty cycles of 15 s @ 95 °C then 45 s @ 56 °C. Primers were for PSEN2 (F: TCCTCAACTCCGTGCTGAAC; R: GCAGCGGTACTTGTAGAGCA), ATXN2 (F: TAATGACGACACAGCCACCC, R: TAGGGGAAATGCGCTGTTGT); CHRNA7 (F: CGGCAAGAGGAGTGAAAGGT, R: AGGCCATAGTAGAGCGTCCT); HNRNPA1 (F: GATCCAAACACCAAGCGCTC, R: CCTTGTGTGGCCTTGCATTC); PICALM (F: GCCAAACTCCCACCTAGCAA, R: TGGTTCCATTTCCGATGCCA)).

#### Mass Spectrometry

Vesicular fractions (fractions 7–9) were pooled and subjected to SDS-PAGE alongside equivalent total amounts of cellular protein. Electrophoresis was terminated once all sample had entered the resolving gel, such that proteins were present as a single band. Gel tops were stained with Coomassie Blue, destained and the protein band excised from the gel and dehydrated using acetonitrile followed by vacuum centrifugation. Dried gel pieces were reduced with 10 mM dithiothreitol and alkylated with 55 mM iodoacetamide. Gel pieces were then washed alternately with 25 mM ammonium bicarbonate followed by acetonitrile. This was repeated, and the gel pieces dried by vacuum centrifugation. Samples were digested with trypsin overnight at 37 °C. Digested samples were analysed by LC–MS/MS using an UltiMate® 3000 Rapid Separation LC (RSLC, Dionex Corporation, Sunnyvale, CA, USA) coupled to an Orbitrap Elite (Thermo Fisher Scientific, Waltham, MA, USA) mass spectrometer. Peptide mixtures were separated using a gradient from 92% A (0.1% FA in water) and 8% B (0.1% FA in acetonitrile) to 33% B, in 44 min at 300 nl min^−1^, using a 75 mm × 250 μm i.d. 1.7 mM CSH C18, analytical column (Waters, Elstree, Herts, UK). Peptides were selected for fragmentation automatically by data dependant analysis. Data produced were searched using Mascot (Matrix Science UK), against the SwissProt_2018_01 database and validated using Scaffold (Proteome Software, Portland, OR, USA). The mass spectrometry proteomics data have been deposited to the ProteomeXchange Consortium via the PRIDE partner repository with the dataset identifier PXD015255.

#### Data Analysis

All experiments are n = 3 unless otherwise indicated. Graphs were prepared using GraphPad Prism 7 (GraphPad Software, Inc., La Jolla, CA, USA).

## Results

### Small EVs Can be Isolated from iPSC-Derived Neurons

**Fig. 1:**
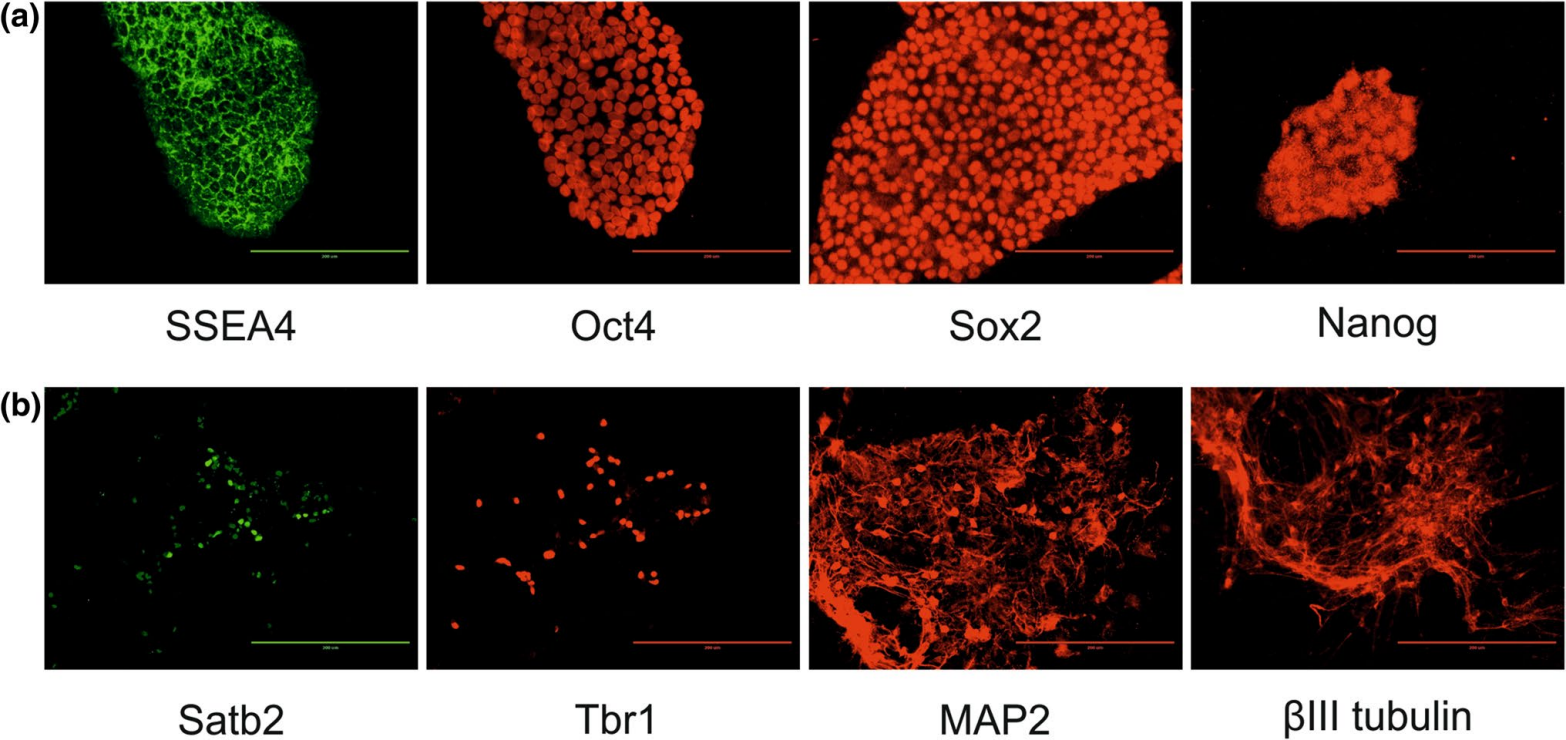
Differentiation of iPSCs to neurons. **a** iPSCs were cultured as described in Experimental Procedures, fixed with 4% paraformaldehyde and immunocytochemistry performed using antibodies against the pluripotency markers SSEA4, Oct4, Sox2 and Nanog. **b** iPSCs were differentiated to neurons as described in Experimental Procedures, fixed and immunocytochemistry performed using antibodies against the neuronal markers Satb2, Tbr1, MAP2 and βIII tubulin. Scale bar = 200 µm

**Fig. 2:**
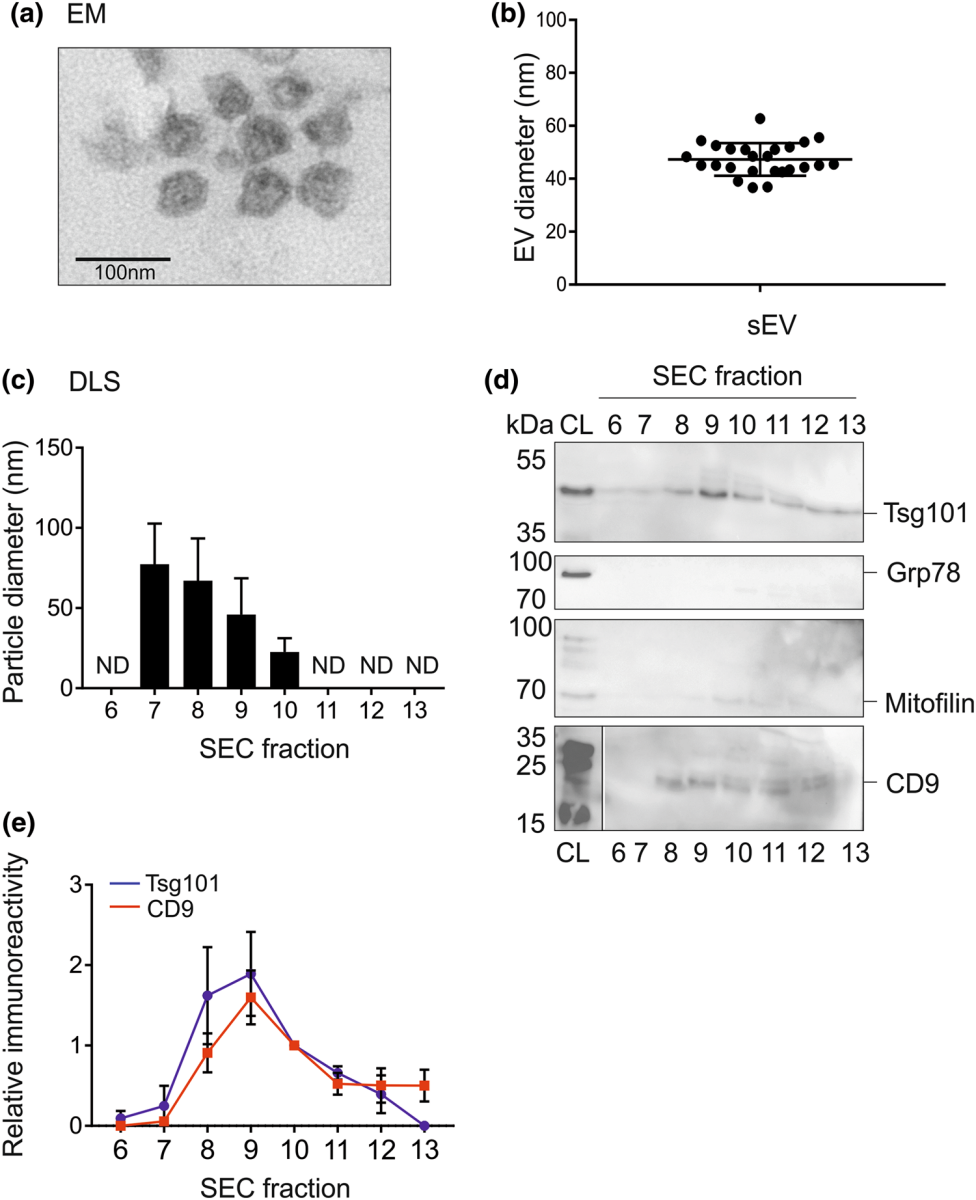
Isolation of EVs from iPSC-derived neurons. iPSC-derived neurons were cultured as described and exosomes isolated from the conditioned medium by size exclusion chromatography (SEC). **a**, **b** The vesicular fractions (7–9) were ultracentrifuged (100,000 g, 90 min) and the pellet fixed and subjected to electron microscopy. Red arrows indicate sEVs **c** EV diameter was calculated from EM images using ImageJ. **d** Unconcentrated SEC fractions (6–13) were assessed for particle diameter using dynamic light scattering. ND = not detected. **e** SEC fractions were concentrated tenfold and subjected to SDS-PAGE alongside cell lysate (CL), followed by immunoblotting for Tsg101, Grp78,mitofilin and CD9. Vertical line in CD9 panel indicates distal lanes from the same gel. **f** Tsg101 and CD9 immunoreactivity was quantified and plotted against SEC fraction number to demonstrate enrichment

Neurons were derived from iPSCs as previously described [25–27]. The differentiated neurons express markers exclusive to mature neurons (including βIII tubulin and Tbr1), are electrophysiologically active from day 49 and form synapses as evidenced by visualisation of protein complex of synaptophysin/ PSD-95 and Munc13/ Homer [27]. Human iPSCs were confirmed as pluripotent (Fig. 1a) and subsequently differentiated into neurons (Fig. 1b and Fig. S1). The conditioned medium from human iPSC-derived neurons was subjected to size exclusion chromatography to isolate EVs. EM analysis confirmed a mean vesicle size of 50 nm (Fig. 2a, b). Dynamic light scattering was performed on individual fractions eluted from the size exclusion chromatography column (Fig. 2c). Fraction 6 represents the final 0.5 ml of the void volume and vesicles were absent from this fraction. Vesicles were detected in fractions 7–10 and the mean particulate diameter decreased between successive fractions, from 77 nm in fraction 7 to 67 nm in fraction 8, 46 nm in fraction 9 and 23 nm in fraction 10 (Fig. 2c). Immunoblotting for the small EV markers Tsg101 and CD9 was performed (Fig. 2d, e). There was minimal immunoreactivity towards Tsg101 in fractions 6 and 7, with overall immunoreactivity highest in fractions 8–10. There was also Tsg101 immunoreactivity in fractions 11–13, which are non-vesicular fractions. Bulk protein was present in these fractions (Fig. S2), which may derive from EVs lysed during isolation or represent proteins present as large extracellular complexes. The pattern of Tsg101 immunoreactivity across the SEC fractions were mirrored by CD9. The ER chaperone Grp78 was not detected in the EV fractions (Fig. 2d). Similarly, there was only very weak immunoreactivity for the large vesicle marker mitofilin (Fig. 2d).

### A Subgroup of Cellular mRNA Transcripts are Selectively Incorporated into Neuronal EVs

**Fig. 3:**
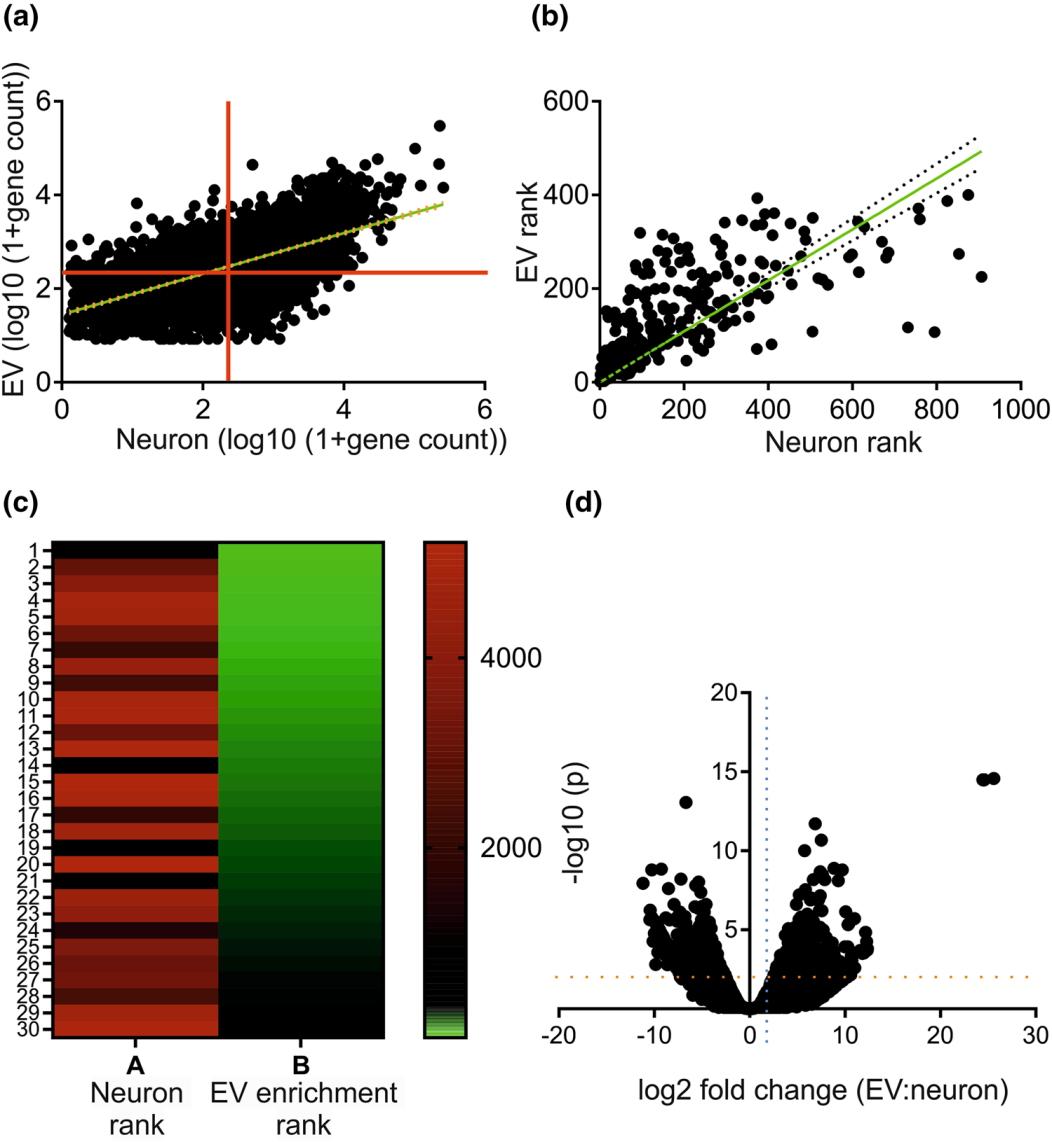
A subgroup of cellular mRNA transcripts are selectively incorporated into neuronal EVs. mRNA was extracted from iPSC-derived neurons (n = 3) and EVs (n = 2), followed by RNA Seq and bioinformatics as described. **a** Abundance of specific mRNA transcripts was compared in neurons and EVs (red line at mean, green line shows linear regression), followed by **b** rank comparison for the 500 most abundant mRNA transcripts in EVs (green line shows linear regression, dotted line shows 95% confidence) and **c** comparison of the top 30 most enriched mRNA transcripts in EVs against their rank abundance in neurons. **d** Volcano plot of all mRNA transcripts. Blue dotted line indicates the log_2_ fold change analysis threshold (log_2_ fold change > 1) and orange dotted line denotes the –log_10_ (p value) threshold (-log_10_ (p) > 2). n number refers to an independent induction of iPSCs to neurons, from each a sEV preparation was isolated (Color figure online)

In order to characterize the mRNA cargo of neuronal sEVs, RNA-seq was performed on the iPSC-derived neurons and their EVs. Comparison of mRNA transcript abundance between neurons and EVs showed a strong positive correlation, although using mean abundance to divide transcripts into quadrants showed a population of transcripts whose abundance was enriched in EVs (upper left quadrant; Fig. 3a). Taking the 500 most abundant EV mRNA transcripts and plotting them against their rank abundance in neurons confirmed a strong positive correlation, suggesting non-specific uptake of the most abundant neuronal mRNA transcripts into EVs (Fig. 3b). The 30 most enriched EV transcripts were plotted against their abundance in neurons (Table 1). The resultant heat map showed the highly enriched EV mRNA transcripts to be predominantly in the lowest tertile in terms of neuronal abundance (Fig. 3c). A volcano plot showed a typical distribution, identifying those subsets of mRNAs that are significantly increased and those that are decreased in EVs (Fig. 3d). Overall, gene body analysis of the top 200 most enriched transcripts showed no difference in read coverage between neurons and EVs. Analysis of 5′ UTR length, 3′ UTR length, %GC content, predicted G quadruplex abundance and poly(A) tail length showed that only %GC content is correlated with EV enrichment. Those genes with the highest fold change between EV and neuron had significantly higher GC content (Fig. S3). These data indicate that the most highly enriched mRNAs incorporated into neuronal EVs are characterized by relatively increased GC content.

**Table 1:** Top 30 most enriched genes in EVs and rank expression in neurons

Gene	Neuron rank	EV enrichment rank	mRNA length (bases)
CCDC9	148	1	2027
KCNE3	3011	2	3143
RASSF3	3983	3	3507
ABCA4	4897	4	7328
SLC14A2	4804	5	4073
TNFSF4	3222	6	3492
SVEP1	2104	7	12,205
CTXN1	4524	8	1237
SNAI2	2352	9	2180
CYLC2	4922	10	2149
SEC14L3	5011	11	2086
SLC6A2	3138	12	2471
MX2	5120	13	3408
ANP32B	224	14	1483
KNG1	5209	15	4198
TNN	5040	16	5042
PRPH	1912	17	1800
ITIH5	4734	18	6716
PLEKHA4	434	19	3073
CLDN11	5196	20	2169
KIF1C	474	21	7917
CHIT1	4595	22	2248
CPNE9	4179	23	2042
C6orf141	1475	24	1450
PAQR7	3633	25	3297
SCLY	3142	26	2526
TRDN	3354	27	1294
FBF1	2437	28	3626
GALNT6	4745	29	5307
CENPP	5064	30	2570

Using RNA seq data, ratios of mRNA abundance were calculated (EV: neuron) and those mRNA transcripts with the highest enrichment were tabulated and compared to their rank abundance in neurons

**Fig. 4:**
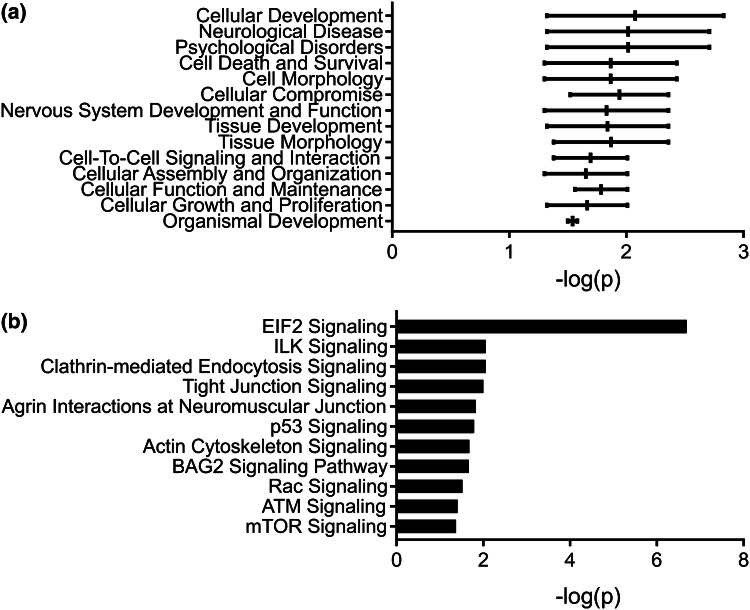
Developmental signalling networks are enriched in the mRNA from neuronal EVs. Following RNA-seq analysis, mRNA transcripts enriched in neuronal EVs (p < 0.01) were processed by Ingenuity Pathway Analysis (IPA) and reported by **a** physiological function and **b** canonical signalling pathways. The horizontal bar indicates the range, where the central vertical line indicates the mean

To elucidate potential functional roles of the most highly enriched neuronal EV mRNAs, Ingenuity Pathway Analysis (IPA) was used. Initially, physiological functions were probed, which revealed a strong focus on development and morphology, with ‘Cellular Development’ and ‘Neurological Disease’ featuring prominently (Fig. 4a and Table 2). Further analysis focusing on canonical signalling pathways showed EV-enriched mRNA transcripts to be linked to ‘Agrin Interactions at the Neuromuscular Junction’, ‘Clathrin-mediated endocytosis’ and various signalling pathways (‘ATM Signaling’, ‘mTOR Signaling’, ‘actin cytoskeleton Signaling’, ‘BAG2 Signaling’ and ‘Integrin-linked kinase (ILK) Signaling’ (Fig. 4b and Table 3). Although IPA outputs are based on experimentally validated data in primary neurons, brain or neuroblastoma cells, this does not imply that these processes are exclusive to neuronal EVs.

**Table 2:** Ingenuity pathway analysis (IPA) gene lists (highly enriched EV mRNA transcripts were selected (q value < 0.05) and assigned to physiological)

Function	− log_10_ p value	Genes
Cellular development	1.32–2.83	ACHE,ACTN1,BAG1,CEACAM1,CLN3,DGCR8,DISC1,EMC10,ERBB3,FMR1,GLIPR1,HIPK2,HNF4A,KNG1,LHX4,LIMK1,MEF2A,NOP53,NOS1,PDGFA,PFN1,PIAS2,PPP1R9B,PTPN11,RUNX1,SCLY,SLC9A3R1,THRB,UNC13A
Neurological disease	1.32–2.71	ACTA2,AEBP1,ASPA,ATF3,BAG1,BBC3,BCAS1,CCDC80,CCN2,CD44,CHRNA7,CIC,CLN3,CLN5,ERBB3,JUN,KNG1,LAMB1,LAMP1,LHX4,MDM2,MECOM,MYO15A,MYO1B,NOS1,PFN1,PIAS2,PRPH,PTPN11,RIPK2,RPL13,RSF1,S100A10,SCLY,TPT1,TRAK2,TRIB3,TRIM56,UNG,VEGFB,VIM,WWTR1
Psychological disorders	1.32–2.71	AEBP1,ATF3,BBC3,BCAS1,CCN2,CD44,CLN3,JUN,KNG1,LAMB1,LAMP1,MYO1B,NOS1,PFN1,PIAS2,PRPH,RIPK2,RPL13,S100A10,TPT1,TRAK2,TRIB3,VIM,WWTR1
Cell death and survival	1.30–2.43	ATF3,CD44,ERBB3,HIPK2,JUN,NOS1,PRPH,PTPN11,RIPK2,RUNX1,SCLY,SH3KBP1,SNAI2,UNG
Cell morphology	1.30–2.43	ACHE,ATF3,CABP4,CHRNA7,CLN3,CLN5,DISC1,DOCK10,ERBB3,FMR1,GATA3,GDF11,HHAT,HIPK2,HSPA4,LHX4,LIMK1,LMNA,MDM2,MSRB3,MYO15A,NOS1,NYAP1,PARD6A,PFN1,RIPK2,RUNX1,S1PR2,SHANK2,SLITRK5,SNAI2,TGFB3,THRB,TLR7,UNC13A,UTRN,VIM
Cellular compromise	1.52–2.36	ASPA,JUN,PIK3CG,SCLY,TRIB3,UNG
Nervous system development and function	1.30–2.36	ACHE,ATF3,BAG1,CABP4,CHRNA7,CLN3,CLN5,DGCR8,DISC1,DOCK10,ERBB3,FMR1,GATA3,GDF11,HHAT,HIPK2,HSPA4,JUN,KNG1,LHX4,LIMK1,LMNA,MDM2,MECOM,MEF2A,MYO1B,NOS1,NYAP1,PARD6A,PFN1,PIAS2,PTPN11,RIPK2,RUNX1,S1PR2,SCLY,SHANK2,SLC24A4,SLITRK5,TGFB3,THRB,TLR7,UNC13A,UTRN,VEGFB,VIM
Tissue development	1.32–2.36	CEACAM1,DISC1,ERBB3,FMR1,HIPK2,MEF2A,NOS1,PFN1,PIAS2,PTPN11,RUNX1,S1PR2,SCLY,UNC13A
Tissue morphology	1.38–2.36	ACHE,CABP4,CHRNA7,CLN3,CLN5,DISC1,DOCK10,ERBB3,FCMR,FMR1,GATA3,GDF11,HHAT,HIPK2,HSPA4,JUN,KNG1,LHX4,LIMK1,LMNA,MDM2,NOS1,NYAP1,PARD6A,PFN1,RUNX1,SCLY,SHANK2,SLITRK5,TGFB3,THRB,TLR7,UNC13A,UNG,UTRN,VIM
Cell-to-cell signaling and interaction	1.38–2.01	ACHE,CD44,CHRNA7,DISC1,FMR1,HIPK2,KNG1,PFN1,SELP,SHANK2,SLC24A4,SLITRK5
Cellular assembly and organization	1.30–2.01	ATF3,CBX1,CEACAM1,CLN3,DISC1,FMR1,HIPK2,NOS1,NYAP1,PFN1,RIPK2,S1PR2,SLITRK5,UTRN
Cellular function and maintenance	1.56–2.01	ACHE,CEACAM1,CLN3,DISC1,FMR1,HIPK2,NOS1,PFN1,S1PR2
Cellular growth and proliferation	1.32–2.01	ACHE,ACTN1,BAG3,CEACAM1,CHRNA7,CLN3,DISC1,EMC10,ERBB3,FMR1,GLIPR1,HIPK2,HNF4A,KNG1,LIMK1,NOP53,NOS1,PDGFA,PFN1,PPP1R9B,PTPN11,SLC5A8,SLC9A3R1,THRB
Organismal development	1.50–1.58	ACHE,ASPA,ATXN1L,CABP4,CHRNA7,CLN3,CLN5,ERBB3,FMR1,FSCN1,GATA3,GDF11,HSPA4,ID1,JUN,KNG1,LAMP1,LHX4,LIMK1,MDM2,MECOM,MSRB3,NOS1,NYAP1,PPP1R9B,SEMA3B,SHANK2,SLITRK5,THRB,TLR7,VIM,ZFYVE26

**Table 3:** IPA gene list for RNA-seq data (canonical pathways)

Pathway	− log_10_ p value	Genes
EIF2 signaling	6.69	ACTA2,ACTG2,ATF3,PABPC1,PIK3CG,PPP1CC,PTBP1,RPL11,RPL12,RPL13,RPL23,RPL23A,RPL26,RPL37,RPL37A,RPL38,RPS12,RPS15A,RPS17,RPS24,TRIB3
Clathrin-mediated endocytosis signaling	2.06	ACTA2,ACTG2,ARPC2,CLTA,EPN1,FGF23,MDM2,PDGFA,PIK3CG,SH3KBP1,VEGFB
ILK signaling	2.06	ACTA2,ACTG2,ACTN1,FLNB,JUN,KRT18,PIK3CG,PPP2R5A,SNAI2,VEGFB,VIM
Tight junction signaling	2	ACTA2,ACTG2,CLDN11,CNKSR3,JUN,PARD6A,PPP2R5A,RAB13,TGFB3,YBX3
Agrin interactions at neuromuscular junction	1.83	ACTA2,ACTG2,ERBB3,JUN,LAMB1,UTRN
p53 signaling	1.79	BBC3,HIPK2,JUN,MDM2,PERP,PIK3CG,SNAI2
Actin cytoskeleton signaling	1.68	ACTA2,ACTG2,ACTN1,ARPC2,FGF23,KNG1,LIMK1,PDGFA,PFN1,PIK3CG,TLN2
BAG2 signaling pathway	1.67	CHRNA7,HSPA1A/HSPA1B,HSPA4,MDM2
Rac signaling	1.52	ARPC2,CD44,JUN,LIMK1,PARD6A,PIK3CG,PLD1
ATM signaling	1.41	CBX1,H2AFX,JUN,MDM2,PPP1CC,PPP2R5A
mTOR signaling	1.37	DGKZ,FKBP1A,PIK3CG,PLD1,PPP2R5A,RPS12,RPS15A,RPS17,RPS24,VEGFB

**Fig. 5:**
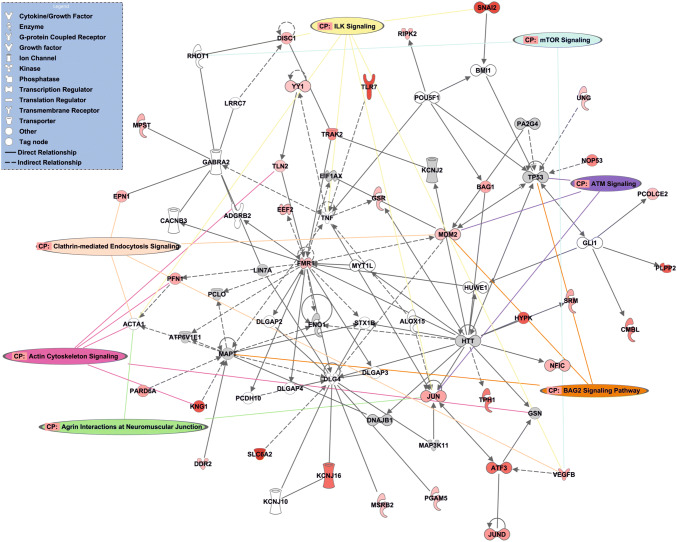
Network analysis of mRNA in neuronal EVs. IPA core analysis was used to generate a network of significantly enriched mRNA transcripts (p = 10^–34^), using 35 focus molecules. Red intensity is proportional to log_2_ fold change, while grey indicates mRNA transcripts that did not reach the analytical cut off (q ≤ 0.01). White coloured molecules indicate network mediators that did not appear in the gene list supplied to IPA. Whole lines indicate direct relationships, while dotted lines indicate indirect relationships. Canonical pathways (CP) are mapped onto the network, connected to their cognate mRNA transcripts by coloured lines. Direct relationships are classed as experimental determined interactions; indirect interactions are predicted based on experimentally determined intermediate interactors (Color figure online)

A transcriptional network was subsequently identified (Cell Morphology, Cellular Assembly and Organization, Nervous System Development and Function) which comprised 35 eV-enriched mRNA transcripts and was highly significant (p = 10^–34^) (Fig. 5 and Table 4). The network formed a hub and spoke structure, where hub transcripts are predominantly transcriptional regulators: *MDM2, FMR1* and *JUN*. (Fig. 5). Furthermore, several of the canonical signalling pathways identified in Fig. 4b mapped onto this network: mTOR signalling, ATM signalling, BAG2 signalling, agrin interactions at the neuromuscular junction, actin cytoskeleton signalling, clathrin-mediated endocytosis and ILK (Fig. 5). In some cases, these canonical pathways were linked by common signalling molecules, specifically *VEGFB* (mTOR signalling, ILK signalling and clathrin-mediated endocytosis signalling), *MDM2* (ATM signalling, BAG2 signalling and clathrin-mediated endocytosis signalling), *JUN* (Agrin interactions, ILK signalling and ATM signalling) and *PFN1* (actin cytoskeleton and clathrin-mediated endocytosis signalling). Together, these analyses show that neuronal EVs contain a putative transcriptional network with key hub genes that connect multiple downstream signalling pathways. In addition, the IPA networks were assessed for inter-connectivity, showing that the networks are predominantly distinct, with few overlapping genes (Fig. S4). Enriched transcripts with links to neuronal function and also dysfunction in neurodegeneration were validated by qPCR: ATXN2, CHRNA7, HNRNPA1, PICALM and PSEN2. The presence of these mRNAs was confirmed in EVs (Fig. S5.)

**Table 4:** IPA network list for RNA-seq data (putative signalling networks generated by IPA)

ID	Function	Focus molecules	Score	Genes
1	Cell morphology, cellular assembly and organization, nervous system development and function	35	34	ACTA1,ADGRB2,ALOX15,ATF3,ATP6V1E1,BAG1,BMI1,CACNB3,CMBL,DDR2,DISC1,DLG4,DLGAP2,DLGAP3,DLGAP4,DNAJB1,EEF2,EIF1AX,ENO1,EPN1,FMR1,GABRA2,GLI1,GSN,GSR,HTT,HUWE1,HYPK,JUN,JUND,KCNJ10,KCNJ16,KCNJ2,KNG1,LIN7A,LRRC7,MAP3K11,MAPT,MDM2,MPST,MSRB2,MYT1L,NFIC,NOP53,PA2G4,PARD6A,PCDH10,PCLO,PCOLCE2,PFN1,PGAM5,PLPP2,POU5F1,RHOT1,RIPK2,SLC6A2,SNAI2,SRM,STX1B,TLN2,TLR7,TNF,TP53,TPH1,TRAK2,UNG,VEGFB,YY1
2	Cell death and survival, cellular assembly and organization, cellular function and maintenance	31	28	Acetylcholine,ACHE,AGER,APC,APP,ARF6,ATF4,BAD,BBC3,BCL2L11,CAMK4,CCDC120,CCL3L3,CCN2,CFL1,CHAT,CHRNA7,CITED2,CLN5,CLTA,COL4A3BP,CRTAP,CXCR4,DDAH1,DNM1L,FKBP1A,FLNA,FOXO1,GLUL,GNAS,GSR,HERC6,HGF,HSPA1A/HSPA1B,HSPA4,HSPB1,IDE,IGF1R,IRAK4,IRF3,IRF7,ITIH5,KCNE3,KIF5B,KIF5C,KLC1,KLC2,LIMK1,MAP2K4,MIF,NENF,NF1,NFKB1,PAK1,PLD1,PRKCA,RFLNB,RNF144B,RPS6KB1,SH3PXD2A,SLC5A7,STIP1,SUMO2,TGFB3,THRB,TPT1,TRIB3,TSC1,UBQLN1,ZNRF1
3	Cell death and survival, protein synthesis	30	27	ADAM17,AEBP1,APBB1,AXL,BCL2L1,beta-estradiol,BIRC5,CASP8,CAV2,CDKN2A,CLDN11,COL1A1,CYP19A1,DGKZ,EGF,EGFR,ERBB2,FN1,GNRH1,GRB2,HK2,HLA-A,ID1,IDE,IGF1R,IGFBP2,ITGB1,JUN,LAMA2,LAMB1,LEP,LEPR,MIR17HG,MSRB3,MYCN,MYOC,NME3,NOS1,NUMB,PABPC1,PDK1,PLP1,PPP1R9B,PTK2,RARA,RGS5,RHBDF1,RPL11,RPL12,RPL13,RPL23,RPL23A,RPL26,RPL37,RPL37A,RPL38,RPS12,RPS17,RPS24,RPS6,SET,SIDT1,SLC2A1,SP1,STAT3,TGFA,TP73,TSHZ2,TSPAN3,VIM
4	Neurological disease, organismal injury and abnormalities, skeletal and muscular disorders	28	24	ACTA2,AGT,APBB1,ARC,ATXN1,ATXN1L,BAIAP2,CCDC80,CCL2,CD14,CTXN1,CYTH4,DLG2,DRD1,EGR1,EGR2,GAPDH,GDNF,GFAP,GRIA1,GRIA2,GRID2,GRIN2A,GRIN2B,GRM2,H2AFX,HMGB1,HMGB2,IL1B,IL6,L-dopa,LMNA,MAPK1,MAPK3,MXD1,MYD88,MYO1B,NET1,NEUROD6,NGF,NPM3,PDGFA,PGPEP1,PIK3CG,PITPNM3,PKM,PPARGC1A,PRKCG,PTBP1,PTGS2,PTPN4,RAB13,RIMS1,S100A10,S1PR2,SELP,SLC39A14,SLITRK5,SLMAP,SNCA,SRF,SYN1,TH,TIRAP,TLR2,TLR4,TRIB3,UNC13A,WWTR1,YWHAE
5	Cancer, cellular development, organismal injury and abnormalities	28	24	ACSL5,ACTG2,ACTN1,ADAM10,BCL2,BIRC5,C2,Ccl7,CCND1,CD44,CDH1,CDH5,CDK5,CDK5R2,CEACAM1,CLEC2D,CRABP1,CTNNB1,CX3CL1,CXCL2,DGCR8,DUSP1,E2F1,EGR2,ERBB3,FOXM1,GATA3,GDF11,GPRC5A,HES5,HNF4A,ID4,IL1R2,IL6ST,INS,ITGAM,JAG1,KIF1B,KMT2D,LAMA2,MSX1,MYO15A,NF2,NOTCH1,NOTCH3,NRG1,NT5DC1,PAK1,PER3,Pou3f1,POU3F2,PPP1R13L,PRX,PSEN1,RGS10,SLC52A2,SMO,SMOC1,SOCS3,STAT3,SYK,TCF7,TGFB1,TGFBR3,TMEM40,TRPV1,TXNRD2,UTRN,WTIP,YAP1
6	Cellular development, cellular growth and proliferation, nervous system development and function	27	23	ADAM17,BAG3,BDNF,C16orf70,CAMK2D,CASK,Cdkn1c,CLN3,DGCR8,DNAJC21,DNM2,EFNA2,EFNA5,EIF2AK2,ELK1,EPO,FLNB,FSCN1,GNAI1,GRIA1,GRIN2B,HIPK2,HLA-A,HTR1A,HTR2C,Ins1,ITIH3,ITPR1,KIF17,KIF1A,KIF3A,LAMB3,LIN7A,MAG,MAP3K11,MAPK8IP1,MEF2A,MEF2C,NF2,NGFR,NOS1,Nos1ap,NRG1,NUDT16L1,NYAP1,PDE11A,PDGFRA,PDLIM1,PPP1CC,PPP2R5A,PTEN,PTPN11,RAC1,RACK1,RASGRF1,RYR2,S100A9,SEMA3B,SHANK2,SIM1,SIRPA,SIRT1,SLC9A3R1,SLITRK5,SYNPO,TNIK,TPP1,TRAF3IP1,WASF1,YWHAG
7	Cellular development, cellular movement, nervous system development and function	24	19	ANKRD13A,ARPC2,ASPA,BCAS1,BCL2,BEST1,CARTPT,CHD4,CLDN11,CNKSR3,CNP,corticosterone,CREB1,DBH,DGCR8,DOCK10,dopamine,FOXM1,GAB1,GIPC1,GNAS,GSN,HES5,HIPK2,HTR2C,IL6R,IRS2,ISL1,JAG1,KCND2,KCNK6,LAMP1,LIF,LMCD1,MCL1,MCTP1,ME1,MET,MSI2,MYRF,NEUROD1,NR4A2,NRP1,OLIG1,OLIG2,PLCH2,PLEKHA4,POU4F1,PRKCE,PRKCH,PRKCQ,PRL,PROM1,PRPH,QKI,RUNX1,S100A13,SEMA5A,SH3KBP1,SOX10,SOX2,SREBF1,STAT3,TAC1,TAF10,TCF7L2,testosterone,TLR4,TRH,TRPV1

Focus molecules was the number of EV-enriched mRNA transcripts included in the network. Score was the negative exponent of Fisher's Exact test result

### Proteomic Analysis of Neuronal EVs Identifies Enrichment of Cellular Maintenance and Endocytic Signalling Pathways

**Fig. 6:**
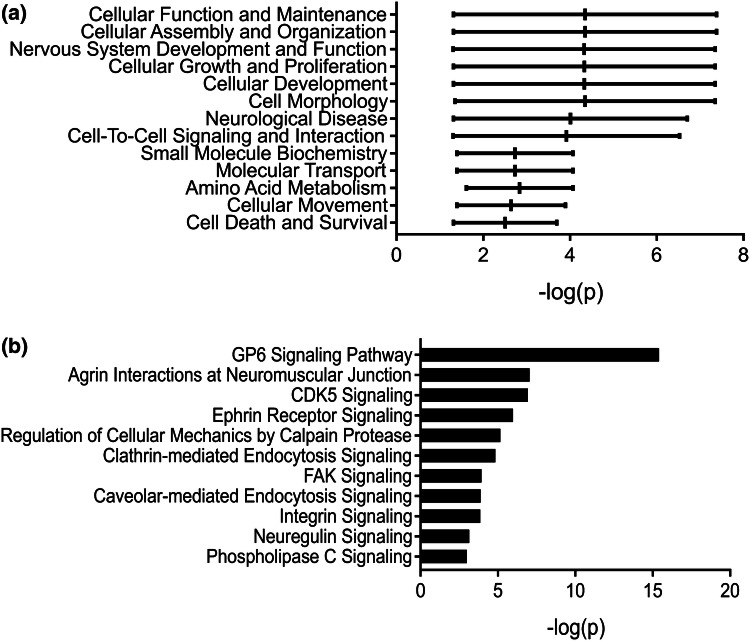
Cellular maintenance and endocytic signalling pathways are enriched in the proteome of neuronal EVs. Following proteomic analysis, proteins enriched in neuronal EVs (total counts ratio > 1 in at least two biological replicates) were processed by IPA and reported by **a** physiological function and **b** canonical pathway. The horizontal bar indicates the range, where the central vertical line indicates the mean

To complement the transcriptomic analysis of neuronal EVs, proteomic mass spectrometry analysis was also performed, followed by IPA on the EV-enriched proteins. Proteomic analysis was able to identify CD9 in the EV sample, but not in the cell fraction. This does not imply absence of expression in neurons, but can be inferred as enrichment in EVs. Several of the IPA categories from this analysis correlated well with the transcriptomic analysis. Common categories included ‘Nervous System Development and Function’, ‘Cell Death and Survival’, ‘Cell-To-Cell Signalling and Interaction’, alongside other morphological and developmental functions (Fig. 6a and Table 5). Investigation of the canonical signalling pathways linked to EV-enriched proteins revealed enrichment of ‘Agrin Interactions at Neuromuscular Junction’ and ‘Glycoprotein VI (GP6) Signalling Pathway’ (Fig. 6b and Table 6). The latter is usually restricted to platelets, but IPA has likely identified this pathway due to substantial enrichment in collagen and laminin isoforms in neuronal EVs. Several signalling and cell entry pathways were also implicated in this analysis: ‘Clathrin-mediated Endocytosis Signalling’ and ‘Caveolar-mediated Endocytosis Signalling’, in addition to ‘CDK5 Signalling’, Ephrin Receptor Signalling’, ‘focal adhesion kinase (FAK) Signalling’, ‘Integrin Signalling’ and ‘Neuregulin Signalling’ (Fig. 6b and Table 6). Together with the transcriptomic data, these proteomic data indicate that neuronal EVs contain proteins that can exert downstream effects via cell surface interactions and also through cell entry. Two proteins were selected for further immunoblot validation, based on their centrality in signalling pathways, either as a master regulator of cell signalling (Src) or a regulator of thousands of RNA targets (TDP-43). Immunoblotting confirmed the presence of both proteins in EVs (Fig. S5).

**Table 5:** IPA gene list for proteomic data (physiological pathways: IPA was also performed using proteomic data and assigned to physiological)

Function	− log_10_ p value	Protein
Cellular assembly and organization	1.32–7.38	FLOT1,VCL,GPC1,GPM6A,LAMB1,LAMC1,ADAM10,A2M,TNR,FN1,ITGB1,TNC,BCAN,SDCBP,LAMA4,LAMB2,RAP2A,PTPRZ1,SRC,LAMA1,SDC2,ATP,LTF,CNTN1,CLU,FAT4,KIDINS220,AGRN,NCAN,RAP1B,HSPG2,APOE,TENM4
Cellular function and maintenance	1.32–7.38	FLOT1,VCL,SYT1,GPC1,GPM6A,LAMB1,LAMC1,ADAM10,A2M,TNR,FN1,ITGB1,TNC,BCAN,SDCBP,LAMB2,RAP2A,PTPRZ1,SRC,LAMA1,SDC2,MFGE8,ATP,LTF,CNTN1,CLU,KIDINS220,AGRN,NCAN,RAP1B,APOE,TENM4
Cell morphology	1.35–7.35	FLOT1,VCL,GPM6A,LAMB1,ITGA2,LAMC1,ADAM10,A2M,TNR,FN1,ITGB1,TNC,SLC1A3,LAMA4,LAMB2,CD81,RAP2A,PTPRZ1,SRC,LAMA1,GPM6B,SDC2,CHMP4B,MFGE8,ATP,LTF,CNTN1,CLU,VCAN,KIDINS220,RAP1B,AGRN,HSPG2,APOE,TENM4
Cellular development	1.32–7.35	FLOT1,TF,VCL,GPC1,GPM6A,LAMB1,LAMC1,ADAM10,A2M,TNR,FN1,ITGB1,TNC,BCAN,SLC1A3,SDCBP,LAMA4,LAMB2,RAP2A,PTPRZ1,SRC,LAMA1,SDC2,ATP,CNTN1,CLU,KIDINS220,VCAN,HBA1/HBA2,AGRN,NCAN,RAP1B,APOE,GPC2,TENM4,NEDD4L
Cellular growth and proliferation	1.32–7.35	FLOT1,TF,VCL,GPC1,GPM6A,LAMB1,LAMC1,ADAM10,A2M,TNR,FN1,ITGB1,TNC,BCAN,SLC1A3,SDCBP,LAMA4,LAMB2,RAP2A,PTPRZ1,SRC,LAMA1,SDC2,ATP,CNTN1,CLU,KIDINS220,VCAN,HBA1/HBA2,AGRN,NCAN,RAP1B,APOE,TENM4,NEDD4L
Nervous system development and function	1.31–7.35	VCL,LAMB1,LAMC1,TNR,TNC,SDCBP,RAP2A,CD81,PTPRZ1,SRC,LAMA1,GPM6B,CHMP4B,LTF,CLU,VCAN,HBA1/HBA2,AGRN,RAP1B,NCAN,HSPG2,GPC2,NEDD4L,FLOT1,TF,SYT1,GPC1,GPM6A,ITGA2,ATRN,ADAM10,LAMA5,A2M,FN1,ITGB1,BCAN,SLC1A3,LAMA4,CXADR,LAMB2,SDC2,COL3A1,MFGE8,ATP,COL18A1,CNTN1,FAT4,KIDINS220,COL2A1,APOE,TENM4,PDCD6IP
Neurological disease	1.32–6.71	ANXA7,TF,VCL,LAMB1,LAMC1,RBP4,COL1A1,KRT14,A2M,TNR,C4A/C4B,FN1,ITGB1,AOX1,BCAN,SLC1A3,LAMA4,LAMB2,CD81,PTPRZ1,SRC,CFI,COL3A1,GPM6B,CHMP4B,H3F3A/H3F3B,ANXA2,MFGE8,ATP,COL18A1,LTF,CNTN1,CLU,COL11A1,VCAN,KIDINS220,AGRN,LPL,RAP1B,HSPG2,COL2A1,APOE,HPX,PDCD6IP
Cell-to-cell signalling and interaction	1.31–6.53	FLOT1,SRC,SYT1,GPM6A,LAMC1,MFGE8,ATP,ADAM10,TNR,CNTN1,FN1,ITGB1,KIDINS220,AGRN,TNC,NCAN,HSPG2,BCAN,APOE,SDCBP,SLC1A3,LAMB2
Amino acid metabolism	1.61–4.07	TNR,SRC,SLC1A3,ATP
Molecular transport	1.39–4.07	A2M,TNR,SRC,SYT1,ANXA2,APOE,ATP,SLC1A3
Small molecule biochemistry	1.39–4.07	A2M,TNR,SRC,ITGB1,ATP,SLC1A3,APOE
Cellular movement	1.39–3.90	PTPRZ1,SRC,LAMA1,SYT1,GPM6A,COL3A1,LAMC1,ADAM10,A2M,TNR,FN1,ITGB1,TNC,SLC1A3
Cell death and survival	1.31–3.70	PTPRZ1,SRC,TF,GPC1,GPM6B,ITGA2,LAMC1,ATP,XPR1,A2M,FN1,CLU,ITGB1,KIDINS220,AGRN,APOE,SLC1A3,LAMA4,PDCD6IP

**Table 6:** IPA gene list for proteomic data (canonical pathways)

Pathway	− log_10_ p value	Protein
GP6 signalling pathway	15.4	COL6A3,LAMA1,COL3A1,LAMB1,LAMC1,COL1A2,COL1A1,ADAM10,COL18A1,LAMA5,COL5A1,COL11A1,RAP1B,COL2A1,LAMA4,LAMB2
Agrin interactions at neuromuscular junction	7.05	SRC,ITGB1,LAMB1,ITGA2,AGRN,RAP1B,LAMC1,RAP2A
CDK5 signalling	6.94	LAMA1,ITGB1,LAMB1,ITGA2,RAP1B,LAMC1,ATP,LAMA5,RAP2A
Ephrin receptor signalling	5.97	MAP4K4,SRC,ITGB1,ITGA2,SDC2,RAP1B,GNB1,SDCBP,ADAM10,RAP2A
Regulation of cellular mechanics by calpain protease	5.16	SRC,VCL,ITGB1,ITGA2,RAP1B,RAP2A
Clathrin-mediated endocytosis signalling	4.84	TSG101,RAB5C,RAB7A,TF,SRC,CLU,ITGB1,RBP4,APOE
FAK signalling	3.94	SRC,VCL,ITGB1,ITGA2,RAP1B,RAP2A
Caveolar-mediated endocytosis signalling	3.88	FLOT1,RAB5C,SRC,ITGB1,ITGA2
Integrin signalling	3.86	TSPAN6,SRC,VCL,ITGB1,ITGA2,RAP1B,TSPAN7,RAP2A
Neuregulin signalling	3.15	SRC,ITGB1,ITGA2,RAP1B,RAP2A
Phospholipase C signalling	2.99	SRC,ITGB1,PLD3,ITGA2,RAP1B,GNB1,RAP2A

## Discussion

As non-coding RNA generally predominates in EVs, the minority RNA subtypes, including mRNA, have not been well studied [32, 33]. There are relatively few reports of RNA-seq in sEVs [33–35] and this study is the first in iPSC-derived neurons. Although there was a strong positive correlation between mRNA abundance in the neurons and their EVs, the most highly enriched EV mRNA transcripts on the whole were less abundant in the neurons, implying that a subgroup of mRNAs are selectively enriched in the EVs. Though mRNA requires translation for functionality, there are several reports of the ability of mRNAs to enter and be translated in recipient cells [7, 8, 32]. In agreement with previous reports, our RNA-seq analysis highlights the large number of mRNA transcripts encoding ribosomal subunits enriched in the neuronal EVs [34]. This may indicate that EVs contain mRNA encoding elements of the translational machinery to facilitate expression of EV mRNA. Other key IPA categories most strongly linked to EV RNA were those involved in cell development and survival. These are well reported functions of EVs [36–40]. Canonical pathway analysis also implicated integrin-linked kinase (ILK) signalling. Integrin signalling is strongly linked to EV function [2, 41] and is likely to play a role in EV-mediated signalling.

Proteomic analysis of the neuronal EVs concurred with the transcriptomic analysis findings; development, cell to cell signalling, morphology and cell growth/ proliferation were also prominent IPA categories. Additional foci of the proteomics analysis were cell interaction and entry pathways, specifically clathrin- and caveolar-mediated endocytosis, both of which have been linked to EV cell entry [42–45]. Integrins were prominent in the proteomic analysis and integrin signalling was a key IPA term. Integrins are strongly linked to EV-cell interactions and cell entry [41, 46]. Lastly, agrin interactions at the neuromuscular junction featured in the IPA for proteins and mRNA, which suggests that neuronal EVs may impact on the organisation of synapses [47].

Overall, the bioinformatics analysis of the neuronal EV transcriptomic and proteomic data segregates into four cellular processes: (i) cell entry pathways and cell surface signalling; (ii) intracellular downstream signalling; (iii) specific functional outcomes (e.g. activation of key signalling pathways); and (iv) general phenotypic outcomes (e.g. ‘Nervous System Development and Function’ and ‘Cellular Organisation and Assembly’). Taking these cellular processes together one could speculate that neuronal EVs interact with neighbouring cells via integrins, collagens, neuregulin and the ephrin receptors and are internalised via clathrin- and caveolin-dependent mechanisms followed by downstream signalling Roles for ephrin receptor, p53, ILK and MAPK signalling have been recently identified for EVs [48–52]. These downstream signalling pathways can lead to synaptic modification through agrin interactions, which may lead to pronounced effects on cellular development and maintenance, aligning with previous reports [53, 54]. These latter effects may be driven, at least in part, by putative transcriptional networks such as the one identified here (Fig. 6).

Although individual iPSC-derived neurons are highly representative of mature human neurons [25–27], there are limitations, such as the uncertain status of epigenetic modifications after reprogramming and maturation [55]. Similarly, cells in this study were cultured in an in vitro 2-D monoculture, which lacks the support of the extracellular matrix and other cell types of the neurovascular unit [56] and the effect of these factors on RNA loading into EVs remains unclear.

In conclusion, exploratory transcriptomic and proteomic analysis of EVs secreted by human iPSC-derived neurons has identified key facets of neuronal EV function, which may link to signalling pathways related to EV entry into cells and key downstream functional effects. This supports previous work showing the ability of EV RNA to enter target cells and modify their phenotype [57–60].

## Electronic supplementary material

Below is the link to the electronic supplementary material.

Supplementary file1 (PDF 1846 kb)

Supplementary file2 (PDF 806 kb)
