# Supplementary material for: Extracellular Vesicles Isolated from Human Induced Pluripotent Stem Cell-Derived Neurons Contain a Transcriptional Network
Source: Neurochem Res. 2020 May 2;45(7):1711–28. doi: 10.1007/s11064-020-03019-w (PMC7297870; doi:10.1007/s11064-020-03019-w)
Supplement: Supplementary file 1 — Supplementary file1 (PDF 1846 kb) [file 11064_2020_3019_MOESM1_ESM.pdf]

Extracellular vesicles isolated from human induced pluripotent stem cell-derived neurons contain a transcriptional network

David A. Hicks[1]*, Alys C. Jones[1#], Nicola J. Corbett[1†], Kate Fisher[1], Stuart M. Pickering-Brown[1], Mark P. Ashe[2] and Nigel M. Hooper[1]*

[1]Division of Neuroscience and Experimental Psychology and [2]Division of Molecular & Cellular Function

School of Biological Sciences, Faculty of Biology, Medicine and Health, University of Manchester, Manchester Academic Health Science Centre, Manchester, M13 9PT, United Kingdom

Current address: [#]Manchester University NHS Foundation Trust, Manchester, United Kingdom; [†]Discovery Research UK, MSD, London, United Kingdom

* Correspondence:
David A. Hicks
david.hicks-2@manchester.ac.uk (ORCiD: 0000-0001-6045-1063)

Nigel M. Hooper
nigel.hooper@manchester.ac.uk

Keywords: Extracellular vesicles, neurons, RNA seq, proteomics, cell signalling

Running title: *Extracellular vesicle subtypes and transcriptional networks*

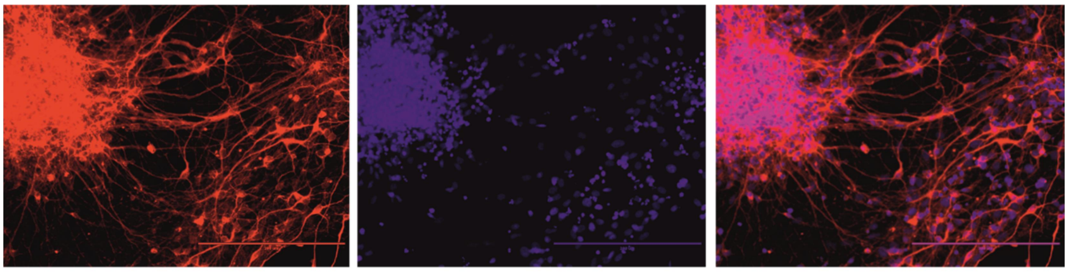

Supplementary figure 1 Differentiation of iPSCs to neurons
iPSCs were cultured as described in Experimental Procedures and differentiated to neurons fixed and immunocytochemistry performed using an antibody against the neuronal marker βIII tubulin and the nuclear stain DAPI. Scale bar = 200μm.

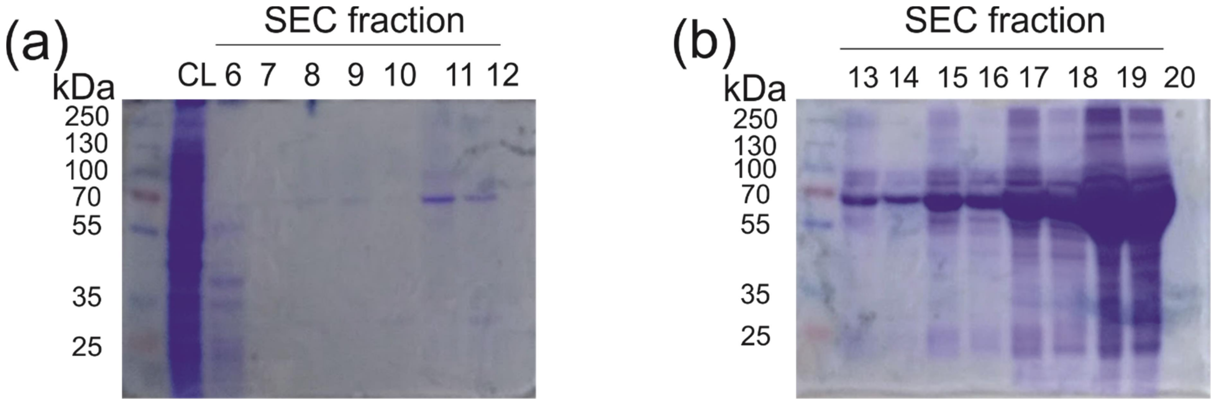

Supplementary figure 2 Bulk protein elutes in non-vesicular SEC fractions
EVs were isolated from iPSC-derived neurons as described by SEC and 0.5 ml fractions collected after elution of the column void volume. These fractions were subjected to SDS-PAGE alongside the cell lysate (CL) and the gels stained using Coomassie Blue. Gels were subsequently destained and imaged.

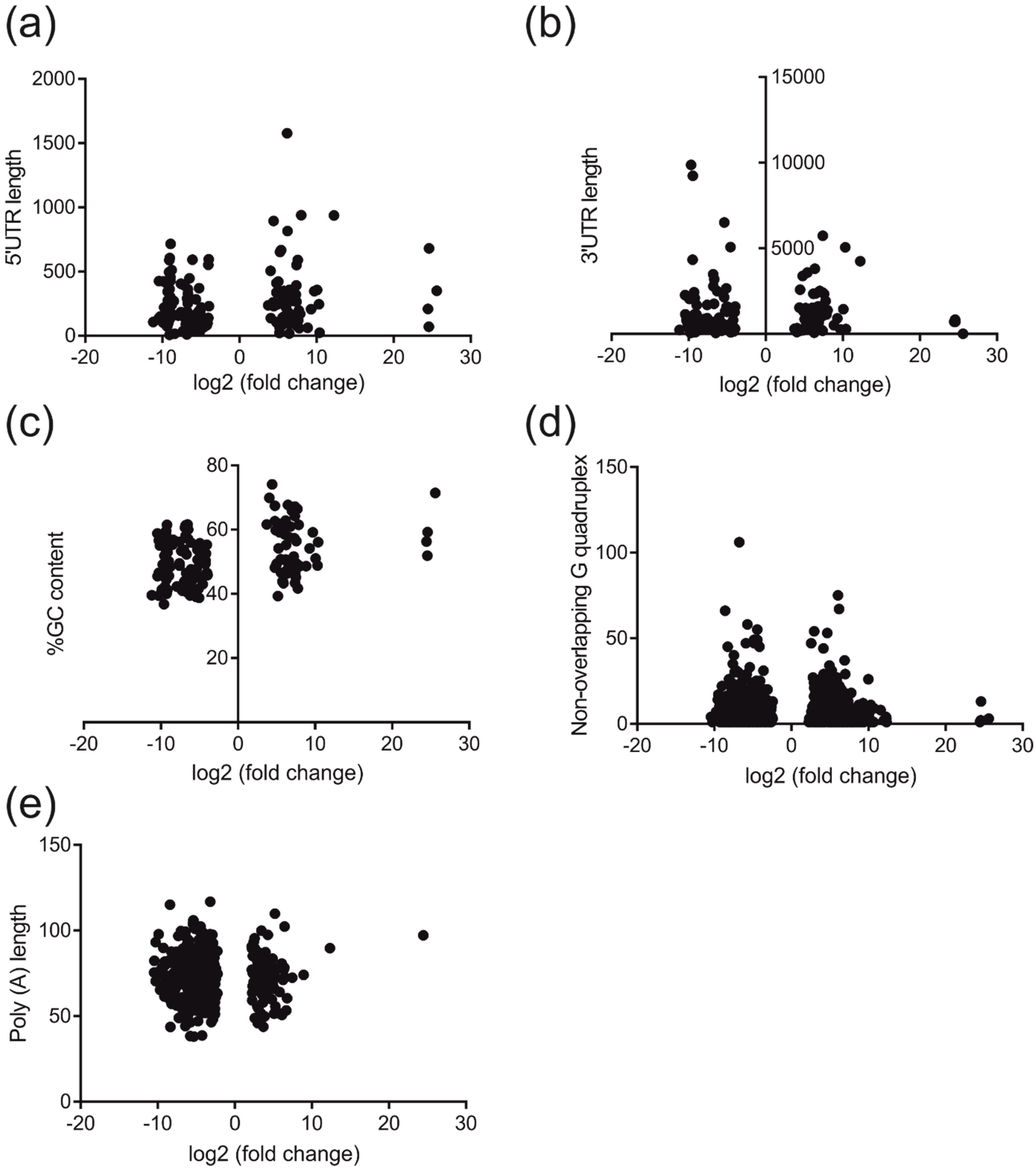

Supplementary figure 3 EV-enriched RNA has a significantly increased GC content

Genes identified by RNA seq were cross-referenced against databases to establish **(a)** 5'UTR length (Spearman r = 0.12; p = 0.15) **(b)** 3'UTR length (Spearman r = 0.09; p = 0.28) **(c)** %GC content (Spearman r = 0.19; p < 0.0001) **(d)** predicted non-overlapping G quadruplexes (Spearman r = 0.03; p = 0.13) and **(e)** poly (A) tail length (Spearman r = -0.02; p = 0.30). Datasets were assessed for Gaussian distribution by D'Agostino Pearson test and correlation by Spearman's rank correlation. Only a subset of the total data for (c) 150/ 18104 total and (d) 500/ 3863 total are displayed.

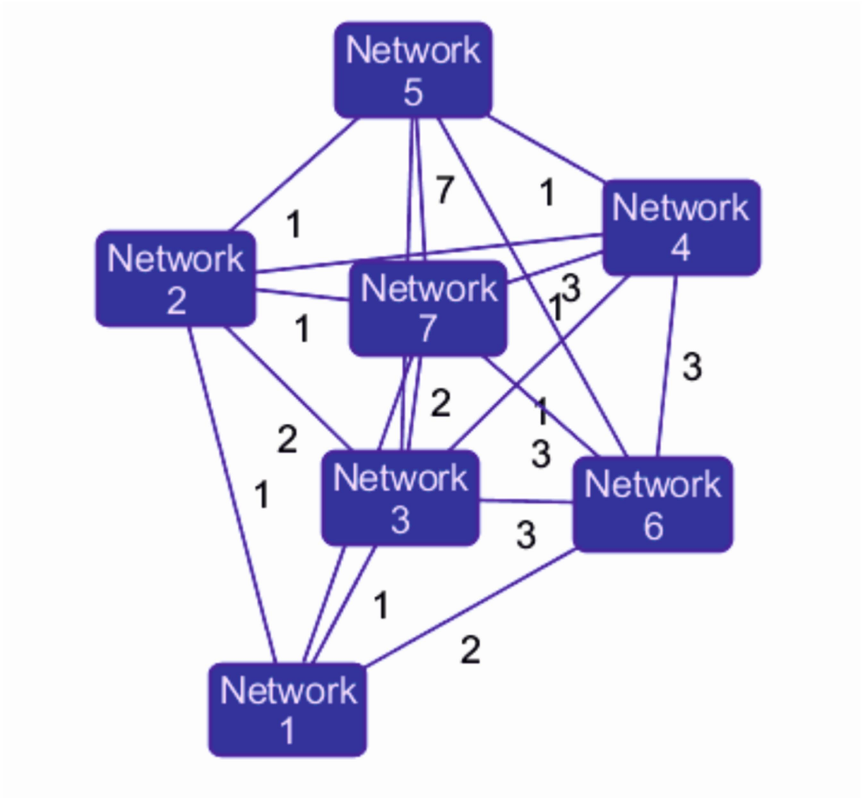

Supplementary figure 4 Transcriptomic IPA networks are predominantly non-overlapping

Transcriptomic IPA networks were displayed to identify gene interrelationships, with common network genes enumerated and shown on inter-network connecting lines.

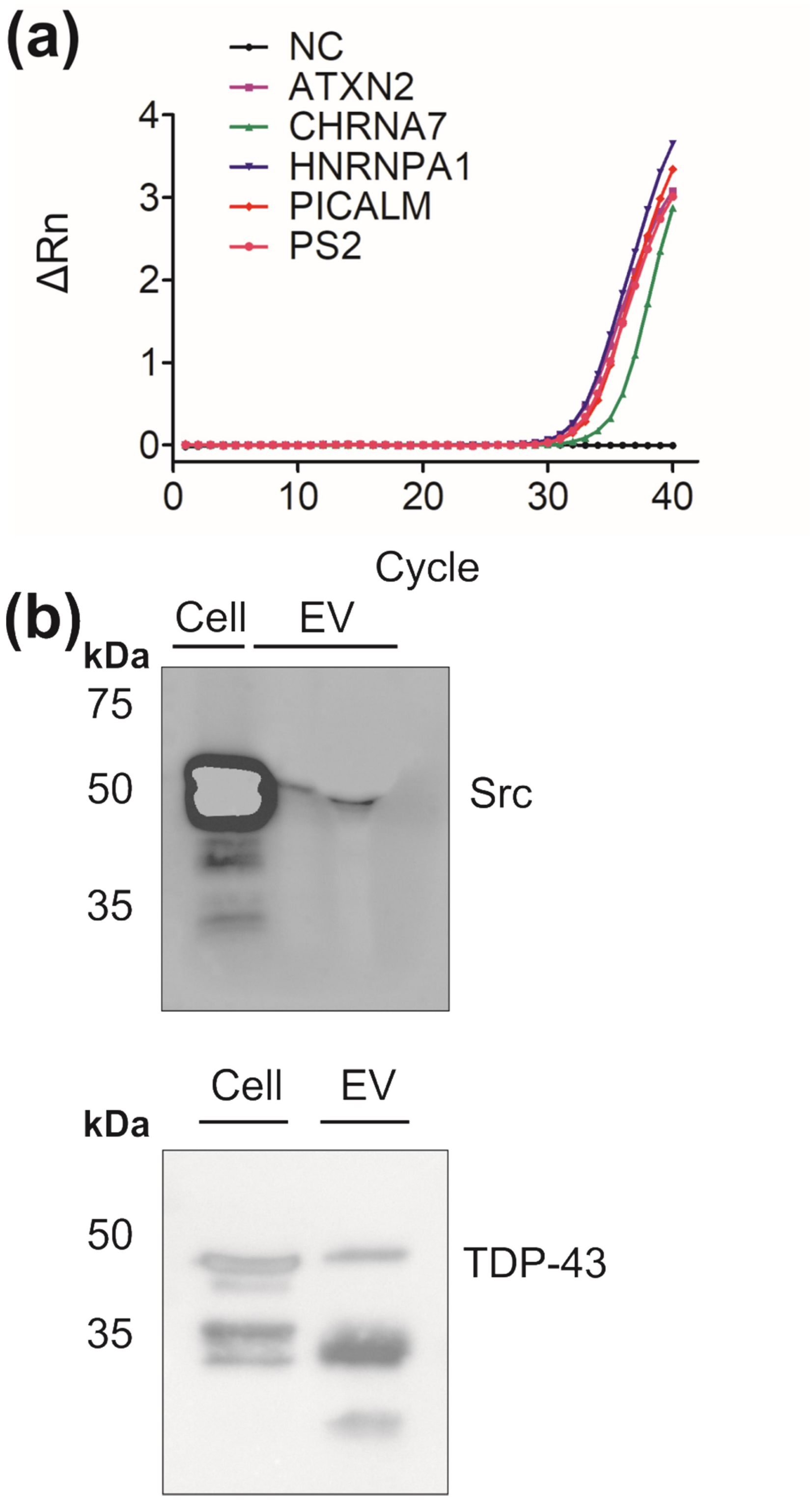

Supplementary figure 5 RNA seq and proteomics data can be validated by qPCR and immunoblot
(a) EV mRNA was isolated as described and the presence of target transcripts assessed by gene-specific primers for ATXN2, CHRNA7, HNRNPA1, PICALM and PSEN2. NC is negative control, with reverse transcriptase omitted from the cDNA synthesis reaction. **(b)** EVs were isolated as described and the vesicular fractions

pooled. Cell and EV samples were separated by SDS-PAGE followed by immunoblotting with antibodies targeting Src and TDP-43.
