# Supplementary material for: Extracellular Vesicles Isolated from Human Induced Pluripotent Stem Cell-Derived Neurons Contain a Transcriptional Network
Source: Neurochem Res. 2020 May 2;45(7):1711–28. doi: 10.1007/s11064-020-03019-w (PMC7297870; doi:10.1007/s11064-020-03019-w)

CD9 original immunoblot

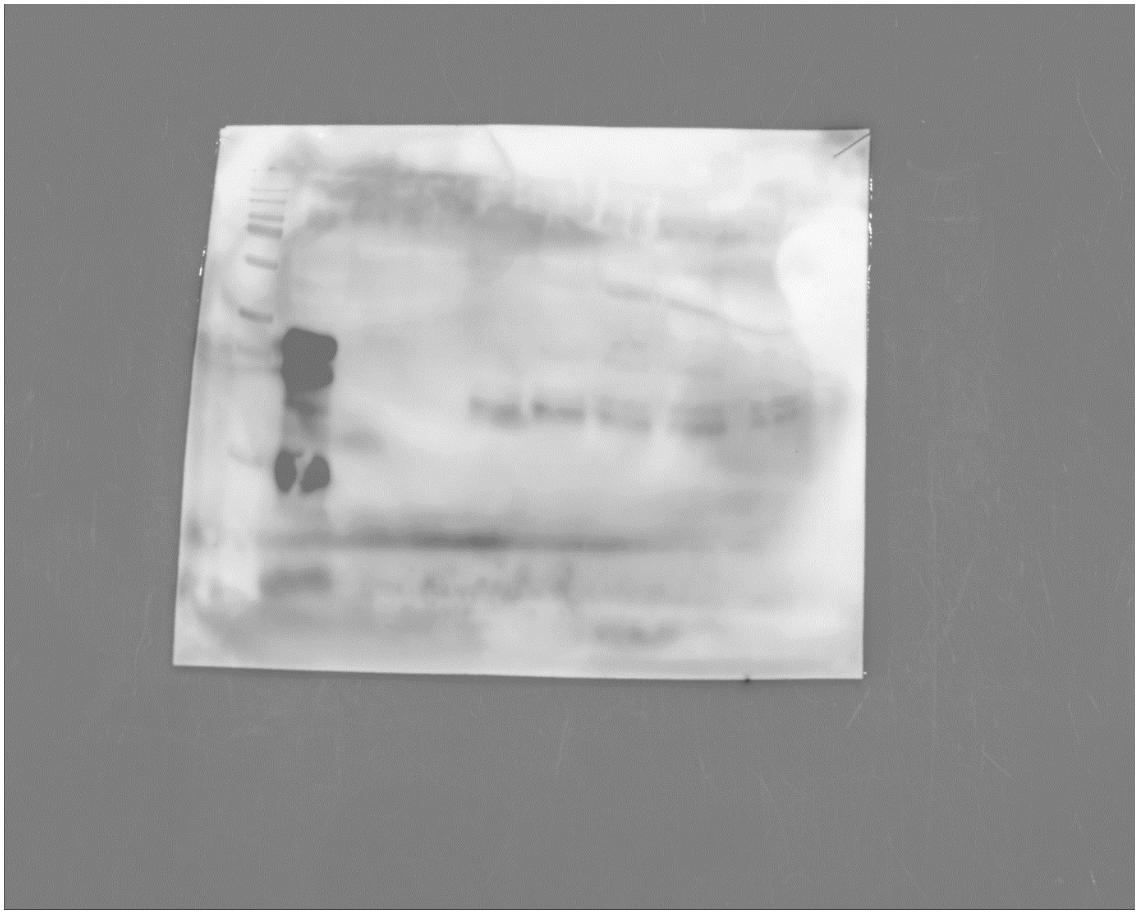

Tsg101 original immunoblot

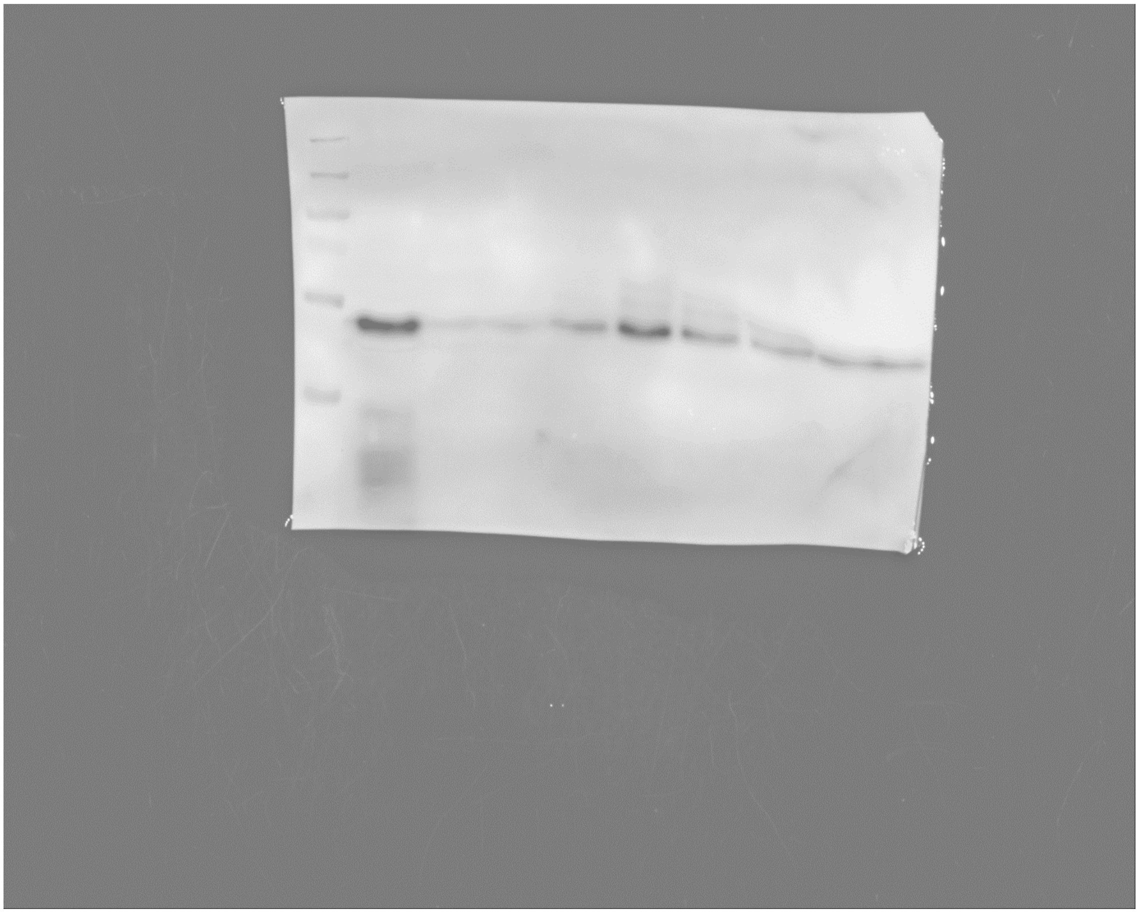

Grp87 original immunoblot

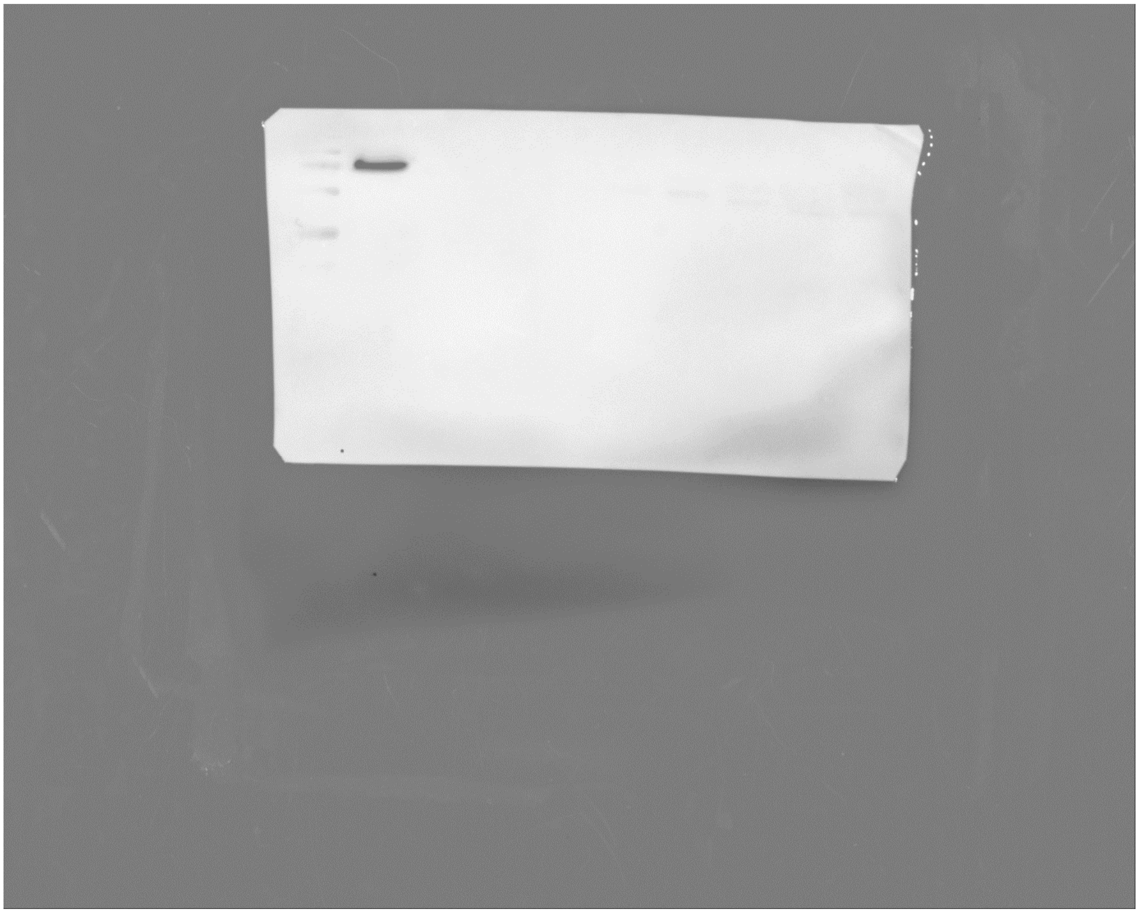

Mitofilin original immunoblot

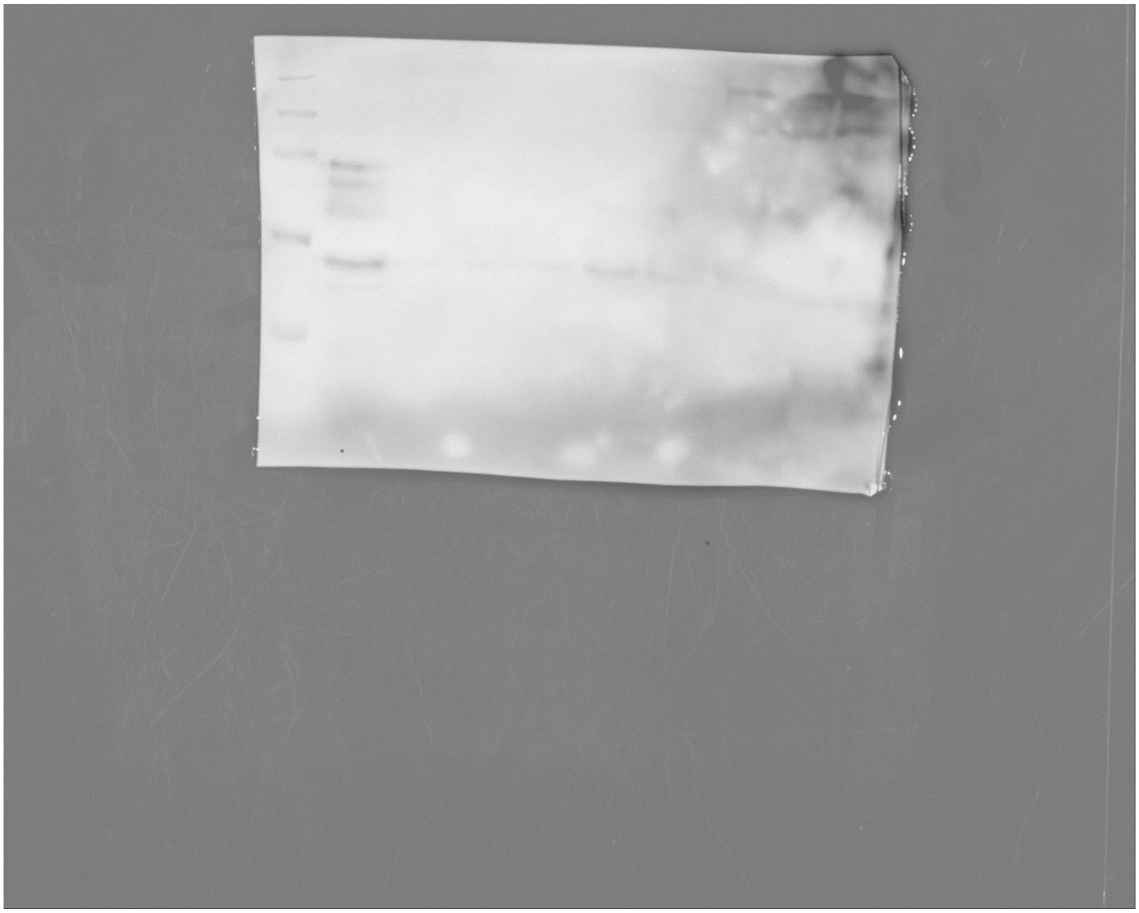

Supplement: Supplementary file 2 — Supplementary file2 (PDF 806 kb) [file 11064_2020_3019_MOESM2_ESM.pdf]
